# Enrichment activities in the medical school psychiatry programme – could this be a key to engaging medical students in psychiatry? A study from a high secure forensic psychiatric UK hospital

**DOI:** 10.1186/s12888-017-1236-z

**Published:** 2017-03-16

**Authors:** Anna-Marie Mortlock, Ignazio Puzzo, Sophie Taylor, Veena Kumari, Susan Young, Samrat Sengupta, Mrigendra Das

**Affiliations:** 1 Broadmoor Hospital, West London Mental Health Trust, Crowthorne, UK; 2 Kings College London, London, UK; 3 Research and Development, Sovereign Health Group, San Clemente, California USA; 4 Centre for Mental Health, Faculty of Medicine, Imperial College London, London, UK; 5 Top End Mental Health Service, PO Box 140, Parap, NT 0804 Australia

**Keywords:** Medical student, Attitude, Psychiatry, Career, Enrichment

## Abstract

**Background:**

The majority of research studies on medical student attitudes toward psychiatry focus on influencing factors and the medical school experience. This study evaluates the effectiveness of a one-day visit to a high secure forensic psychiatric unit on medical students’ attitudes towards psychiatry and also assesses career intentions and the factors influencing these.

**Method:**

Change in attitudes and career intention were measured by administering a questionnaire, which included the 30-item Attitudes Toward Psychiatry (ATP-30) survey, at the start (time 1) and end (time 2) of the one-day visit. Qualitative data on factors influencing career choice was also gathered.

**Results:**

Evaluation of 284 responses revealed a significant increase in positive attitude towards psychiatry from time 1 to time 2 in the sample as a whole. The most influential factor on consideration of psychiatry as a career across all groups was the medical school clinical placement. For those that tended away from choosing psychiatry as a career, patient prognosis was important.

**Conclusions:**

Poor recruitment in psychiatry in the UK is already established which will doubtless be compounded by controversies surrounding the proposed new junior doctors’ contract. Now more than ever, the need to inspire and motivate those at medical school encountering psychiatry is crucial. Our findings add to the body of evidence that the medical school clinical attachment is fundamental in shaping attitudes. However, these results also show that a well-planned visit to a specialised psychiatric unit outside of traditional placements can have a significant impact on students’ attitudes toward psychiatry and mental illness in general. There is limited literature in the UK on enrichment activities within the psychiatry medical school curriculum. We propose that developing opportunities for enrichment activities within psychiatry could increase the scope of how we engage students in this fascinating field of medicine.

**Electronic supplementary material:**

The online version of this article (doi:10.1186/s12888-017-1236-z) contains supplementary material, which is available to authorized users.

## Background

Psychiatry is not looked upon favourably as a prospective career choice by most medical students [1] and for many years psychiatry has failed to attract adequate recruits both in the UK and elsewhere [2]. As a career of first choice, one and three years after qualification, only 4–5% of doctors chose psychiatry [3]. In 2011 and 2012, only 83% and 85.3% of Core Trainee 1 posts (first year training), respectively, were filled following a second round of recruitment [4].

In what is increasingly becoming a struggling system, mental health care in the UK is now firmly on the political agenda [5] and awareness of this crisis is growing. With recent developments in the UK related to a proposed controversial new junior doctors’ contract, addressing the shortfall of those specializing in psychiatry is now more pressing than ever [6, 7].

In investigating the seeming disregard of the specialty, firstly it has been found that perceptions of psychiatry are influenced by stigma [2, 8]. Who can deny the often negative portrayal in film and media [9]? A recent study suggested that the stigmatization of psychiatry discourages medical students from pursuing the specialty as a career [2]. Moreover, this deprecation may even originate from fellow medical students, doctors in other specialties and even staff on the psychiatric wards themselves [10]. Secondly, psychiatric practice is regarded by many as having a lack of scientific basis [4, 11]. Finally, students express frustration in the context of treatment effectiveness [10, 11]; that patients cannot be “fixed” or “cured”.

Deeper exploration of the literature reveals those with a more favourable attitude towards psychiatry tend to be female [12] and those with prior experience of the subject [13]. When combined, Kuhnigk et al. [13] found that students who were both female and had psychiatry/psychotherapy experience had an attitude that was *significantly* more favourable. Moreover, personal experience or a family history of mental illness has been found to be associated significantly with choosing psychiatry as a career [1, 13]. Conclusions on ethnic differences are more inconsistent however. One study reported that Chinese and South Asian medical students demonstrated more stigmatizing attitudes toward delusions and hallucinations than their white British counterparts [14] although later research found that Chinese students (in China) reported positive attitudes towards psychiatry, openness with regard to psychiatric services and respect for psychiatric patients [8].

Intended to serve as an exciting introduction to the specialty, medical school clinical attachments in psychiatry often reinforce misconceptions through negative experience, leaving students with the bitter recollection of feeling under-prepared and even intimidated by patients [10]. Not surprisingly a positive attitude towards psychiatry has been found to correlate with an intention to pursue it as a career [10, 12, 13, 15]. In a recent study, a single day visit was shown to be effective in altering the attitudes of medical students towards forensic psychiatry within a high-security psychiatric hospital [16].

In this study, we report on the factors influencing a medical students’ decision with respect to psychiatry as a career choice adding to a growing body of evidence. However, we also present new findings that highlight the importance of a positive clinical experience in the form of an enrichment activity outside of traditional psychiatry teaching settings.

## Method

This was a quantitative and qualitative survey of data collected from medical students (*n* = 289) on one-day visits to Broadmoor high-secure forensic psychiatric hospital. Broadmoor hospital, which has 210 beds, treats and rehabilitates the highest risk mentally disordered offenders and is one of three high secure hospitals in England. The study was registered as a service evaluation of the hospital’s medical education programme.

### Participants

Participants comprised third and fourth year medical students at Oxford University, Imperial College-, St George’s- and King’s College University of London medical schools. Students attended as part of compulsory training.

### The visit

Groups comprised between 10 and 20 students and the visit was hosted by a consultant psychiatrist and a trainee in psychiatry. The day began with a question and answer session, followed by 1.5 h on the ward where students interviewed between one and five patients under supervision. There was then a tour of the hospital and a 2-h teaching session on forensic psychiatry. The visit lasted approximately 6 h and took place weekly over a period of 9 months.

All students were asked to complete a paper questionnaire at the beginning and end of the visit. Students were told that they had the option not to complete the questionnaire. They were each allocated a number to maintain anonymity and they were not told that they would be answering the questions again in the afternoon.

### Measures

From existing literature [1–4, 10, 17] a questionnaire was designed to ascertain medical students’ attitudes towards psychiatry, their likelihood of pursuing psychiatry as a career and factors that may influence this decision (see Additional file 1 which shows this in more detail). Data on gender, age and ethnicity was also collected, as well as having any qualification and/or experience of psychiatry or working in mental health prior to medical school.

Intention of considering a career in psychiatry was measured using a 5-item rating scale ranging from ‘definite intention not to consider’ to ‘definite intention to consider’. Based upon literature review, a set of factors were then presented and the students invited to identify those they felt had influenced this decision. These factors consisted of medical school clinical placement experience, experience in a mental health setting outside of medical school, perception of psychiatry by the public, perception of psychiatry by the medical profession, perceived lack of evidence base, factors related to prognosis of patients and career opportunities. Students could pick more than one factor. There was also an opportunity for a free-text response from which themes were drawn.

Students’ attitudes to psychiatry were measured using the 30-item Attitudes Towards Psychiatry (ATP-30) scale [18]. This scale has been used in numerous studies [4] both nationally and internationally to assess the impact of teaching and clinical attachments [13, 19, 20] and shown to be sensitive in detecting positive shifts in attitude after students had clinical psychiatric attachments [4]. It uses a 5-point Likert scale that consists of questions about attitude to psychiatric patients, illness and treatment, psychiatrists, psychiatric institutions, teaching, knowledge and career choice. It generates a global score between 30 and 150, with higher scores indicating more favourable attitudes. The ATP-30 has been demonstrated to have good face validity, concurrent and construct validity, split-half reliability and high test-retest correlation [18].

### Data analysis

The questionnaire answers were inputted, concurrently with data collection, into an electronic database using the Statistical Package for the Social Sciences (SPSS) version 22 (IBM Corp., USA) (Additional file 2).

We computed the change in score for each individual participant as a percentage using the following formula ((ATP-time2 – ATP-time1)/ATP-time1)*100. Using this formula we created a new variable showing the percentage change in attitude towards psychiatry, whereby a positive value indexes an increase in attitude (positive change) towards psychiatry between time 1 and time 2 and a negative value indexes a decrease in attitude (worsening of attitude) towards psychiatry between time 1 and time 2.

A paired-sample *t*-test was performed in order to assess for any change in attitude towards psychiatry between time 1 (beginning of the one-day visit) and time 2 (end of the one-day visit). A multiple linear regression model was implemented to ascertain which data variables (if any) were predictors of positive change in attitude.

The free-text responses were subjected to thematic qualitative analysis. The first step of the thematic analysis was to read through all of the respondents’ answers. Then the responses were re-read to identify strings of text expressing a single meaningful thought. Next these were grouped into themes. Key themes that tended to re-occur were noted.

## Results

Data collection took place between August 2014 and May 2015. Of 289 students in total, 35 students’ responses were excluded due to not being adequately completed yielding a response rate of 88%. Of the remaining 254 students (110 males and 144 females), 204 had an age range of 18 to 24 years and the remainder were 25 years or older (Table 1).

**Table 1:** Socio-demographic characteristics of the participants

	*n* (%)
Gender	
Male	110 (43)
Female	144 (57)
Age (yrs)	
18–24	204 (80)
25+	51 (20)
Ethnicity	
Caucasian	133 (53)
Asian	82 (32)
Black	19 (8)
Mixed	19 (8)

The majority of students had not had any formal experience in mental health prior to medical school (86%) or any previous qualifications (65%). Most had had a medical school clinical placement in psychiatry (87%) (Table 2).

**Table 2:** Medical students’ experience prior to and during medical school

	*n* (%)
Higher training e.g. previous degree/qualification prior to medical school	
Yes	77 (35)
No	144 (65)
Clinical attachment in psychiatry during medical school	
Yes	218 (87)
No	34 (13)
Work experience/employment in mental health prior to medical school	
Yes	32 (14)
No	204 (86)

Only 15 students expressed a definite intention to consider psychiatry as a future career (6%) and 33 students (13%) were ‘more likely’ to consider a career in psychiatry. In contrast, 81 students (32%) were ‘less likely’ to consider psychiatry; 40 students (16%) were ‘definitely not’ inclined towards the specialty and 85 students (34%) were undecided.

Across all groups except for the ‘Definitely yes’ subgroup, clinical attachment experience was the most commonly cited factor influencing career choice (Table 3). The ‘Definitely yes’ subgroup, was the only subgroup for which clinical attachment experience was not the most frequent response, with 80% of students (*vs*. 73% for clinical attachment) opting for career opportunities.

**Table 3:** Medical students’ responses to the questionnaire item ‘Factors influencing decision’ when considering a career in psychiatry

	Definitely not	Less likely	Might/might not	More likely	Definitely yes
	*n* = 40 (%)	*n* = 81 (%)	*n* = 85 (%)	*n* = 33 (%)	*n* = 15 (%)
Clinical attachment experience	27 (68)	60 (74)	79 (93)	31 (94)	11 (73)
Experience outside of medical school	7 (18)	14 (17)	32 (38)	15 (45)	9 (60)
Public perception of psychiatry	10 (25)	10 (12)	24 (28)	17 (51)	5 (33)
Perception of psychiatry within medicine	12 (30)	22 (27)	37 (44)	15 (45)	4 (27)
Perceived lack of evidence base	10 (25)	21 (26)	17 (20)	4 (12)	2 (13)
Patient prognosis	20 (50)	42 (52)	40 (47)	13 (39)	2 (13)
Career opportunities	16 (40)	18 (22)	41 (48)	19 (58)	12 (80)
Other	8 (20)	13 (16)	7 (8)	7 (21)	5 (33)

Free-text responses were submitted by 40 students. Of the 40 responses, 21 were from students who were ‘definitely not’ or were ‘less likely’ to consider a career in psychiatry and 12 were from those who were ‘definitely yes’ considering or were ‘more likely’ to consider a career in psychiatry. Of the 21 students tending away from psychiatry, a prominent theme (endorsed by 10 of 40) was that another speciality was already preferred (paediatrics was cited twice and neurology once) and 4 expressed concerns about psychiatry being emotionally difficult. One student referred to patients being challenging and another to the subject being slow paced. Interestingly of the 21 students tending away from psychiatry 14 had had a clinical placement and 7 were aged 25 years and over. A theme that emerged for those who were undecided or tending towards a career in psychiatry was interest in the subject (endorsed by 6 students) and lifestyle (endorsed by 4 students) (Table 4). The theme ‘lifestyle’ reflected students who quoted ‘retirement’ and ‘better quality of life’ in their answer. 2 students quoted ‘lifestyle’ but did not expand on this.

**Table 4:** Medical students’ free text responses to ‘Factors influencing decision’ when considering a career in psychiatry

Positive	Negative
*“Psychiatry will become the most in demand speciality in the future”* *“I am interested in the conditions and I think psychiatric patients are some of the most in need yet stigmatised”*	“*It’s slow paced; I think you generally have to wait a long time to see improvement”* *“Little hand-on skills”* *“Higher rates of depression in psychiatrists”* *“Listening to someone’s problems every day and not getting affected by it would be difficult for me”*

Following the one-day visit, we found a positive change in the medical students’ attitudes (Table 5). ATP scores increased significantly between time point 1 (ATP-30 completed at the start of the visit) and time point 2 (ATP-30 completed at the end of the visit); t (254) = 7.312; *p* < 0.0001.

**Table 5:** Paired sample *t*-test of the total ATP-30 scores before and after a one-day visit by medical students to Broadmoor high secure forensic hospital

	Mean	Median	Standard deviation	Mean difference	t-score
Before	110.6706	111	12.78230		
After	113.6314	114	13.28597		
				2.96078	−7.312

In order to explore and better characterize the significant change in attitude towards psychiatry between time 1 and time 2, we used multiple linear regression analysis. Our dependent variable was the percentage change in attitude between time 1 and 2 and our predictor variables were gender, age, ethnicity and clinical placement in psychiatry during medical school (“Experience”). Results showed that these four predictors significantly explained 3.9% (R^2^ = 0.039) of the variance in the change of attitude towards psychiatry: *P* < 0.05 F(4245) = 2.489; *p* = 0.04; *P* < 0.05 (Table 6).

**Table 6:** Multiple linear regression model identifying the variables that were predictors of positive change in medical students’ attitudes

Unstandardized coefficients					
		B	Std. Error		
	(Constant)	1.326	1.668	.795	.428
	Gender	-.186	.776	-.240	.810
	Age	.408	1.009	.405	.686
	Experience	2.419	1.143	2.115	.035*
	Ethnicity	−1.466	.810	−1.811	.071^Trend^

*p= < 0.05

Moreover, the predictor “Experience” contributed significantly to the effect. In fact, results showed that the percentage change in attitude towards psychiatry is *significantly* greater in students that had a clinical placement in psychiatry during medical school compared to students who did not.

We also found a trend towards significance for our ethnicity predictor, suggesting Caucasian students showed a decrease in percentage change compared to students with a non-Caucasian background. Therefore, Caucasians tended to have a negative change in attitude towards psychiatry as compared with non-Caucasians.

## Discussion

We examined the effects of a one-day visit to a high secure forensic psychiatric hospital on medical students’ attitudes towards psychiatry and also assessed career intentions and the factors influencing these.

Our findings show that a one-day visit to a psychiatric unit can positively change attitudes of medical students towards psychiatry in the short-term (without additional assessments, it is impossible to know if such a visit would have long-term effects on attitudes). Having experience already of the subject was found to contribute most to this effect. We further found that the percentage change in attitude towards psychiatry is significantly greater in students that had a clinical placement in psychiatry during medical school compared to those who did not. These findings are supported by previous work showing that clinical attachments in psychiatry resulted in more positive attitudes towards the specialty and increased career interest [17]. It is notable that contact with someone with mental illness, as may occur in clinical settings, was found to be better than education at reducing stigma [15]. Our findings also show that experience gained in mental health settings even outside of medical school is important; ranked third in the ‘definitely yes’ group.

However, the clinical placement was also ranked as the most popular influencing factor for those who tended away from psychiatry. There may be many variables associated with a clinical placement. It has been found both that number and quality of attachments are significantly associated with higher ATP scores [15] and that clinical encounters are specifically pivotal in changing career intentions [4]. Farooq et al. [15] found that the odds of choosing psychiatry dropped to almost half as much for those who had experienced didactic teaching.

One possible reason for the importance of the clinical attachment is in relation to the “role model”. Role models in a particular clinical area are associated with medical students’ choice of clinical field [21]. Sixteen years after this finding, Archdall et al. [10] proposed that medical students were encouraged to pursue psychiatry because of “inspiring, motivating or enthusiastic role models” which may even be more influential than the specialty itself.

The second most popular influencing factor for those tending away from a career in psychiatry was students’ perceptions about patient prognosis. This was found to be the most negatively rated factor in a previous study [2]. Our thematic analysis also revealed that psychiatry was viewed as uninteresting which is supported by previous research [1] and in addition highlighted a perceived emotional burden of the work [10].

It is curious that public perception was not highlighted to the degree that we were expecting for those tending away from psychiatry. In fact, it was the least selected factor and was chosen more in those who were tending *towards* a career in psychiatry. However, it was notable that in those inclined not to choose psychiatry, perception of psychiatry *within medicine* was more frequently chosen than public perception. Indeed, Archdall et al. [10] found that a powerful source of stigma was from other medical students who had already undertaken an attachment in psychiatry. Perhaps, it may be that focus needs to be turned to the views and attitudes held within our own healthcare profession. This has been echoed in previous work [2].

It is also interesting that of those who ‘definitely’ wished to pursue psychiatry, 80% of students chose the career opportunities factor (closely followed by clinical placement; 73% of students). This is in keeping with Goldacre et al. [3] who found anticipated hours and working conditions as being one of three factors having a greater influence on choosing psychiatry as a career. This was also reflected as a theme in our qualitative analysis.

Although we found age did not impact significantly as a predictor of positive change in attitude, there is literature to suggest that maturity of students is a factor in how the psychiatry clinical attachment is experienced [10]. The crude distinction between those below and above the age of 25 in our study likely prevented identifying any age-related trends.

Overall, our study demonstrates that change in attitude can be effected by a one-day visit to a specialised psychiatry unit. This is supported by previous research that found exposure to enrichment activities (special study modules, electives and university psychiatry clubs) increased the likelihood of students choosing psychiatry [15]. However, there is limited literature that focus on enrichment activities within psychiatry in the UK. One study in Canada found a positive effect of a week long summer program for first and second year medical students on subsequent recruitment to a psychiatry training program [22]. In other studies from the US, residents indicated that psychiatry enrichment activities had been important to them, particularly psychiatry electives [23, 24].

Students who only gain their psychiatry experience from the traditional general adult or older adult settings may obtain a skewed view. Offering students the opportunity of an experience in a specialist centre such as a local addiction unit or secure facility can be a potential strength [25]. It may be that by increasing the opportunity for enrichment activities may not only help cultivate interest in psychiatry but help combat stigma and foster improved attitudes.

### Strengths and limitations

The sample size is comparable to other studies and is therefore a strength. In addition, the students attending the visits did so as a compulsory part of their course and were not a self-selecting group. Furthermore, students’ feedback was recorded immediately after the visit and therefore any changes seen can be assumed to have been influenced by the visit itself.

The generalizability of results may be limited as the study took place in a specialised psychiatry unit, but we found that our findings support much of what is known already about students’ experience of psychiatry in particular with regards to clinical attachment.

In addition, the results may have been influenced by differences in the patient group or the hosting doctors between visits. It is also possible that students may have been influenced by a researcher from the study introducing the questionnaire in the session despite the assurance of anonymity. It has been considered that different medical schools may place a different emphasis on psychiatry in both academic studies and clinical placements, however, we attempted to ameliorate anxiety about honestly expressing a negative attitude towards psychiatry by not collecting individual data on which medical school was attended and which year of medical school the students were in. A similar consideration had been made in the study by Burra et al [18]. The expression of both positive and negative views suggested that students felt able to be honest.

In the interests of developing a questionnaire that was brief but useful, a number of areas of interest were not explored which included religious, cultural and other social aspects. In addition, the effect of personal experience or a family history of mental illness on attitudes was not collected. These areas would be important and useful to investigate in development of this work.

Development of this work could also include using a second questionnaire such as the Attitudes to Mental Illness Questionnaire (AMI) or the 6-item Psychiatric Experience Attitudes and Knowledge questionnaire (PEAK-6). Furthermore, experimental conditions could be introduced such as dividing each cohort and exposing each to a different condition, for example, different lengths of time spent with patients.

Although not the primary aim of this study, evidence of an enduring rather than transient change would be useful. It has been shown that whilst improvement in attitude towards psychiatry after a psychiatric attachment diminishes over time, declining over a year [10], it does remain higher than beforehand in newly qualified doctors [12] and 64% who had made their decision in the first year post qualification remained within the field after 10 years [3].

## Conclusions

The influencing factors upon a medical students’ decision to choose psychiatry as a career are varied, nevertheless it seems that experience gained in the subject is key, particularly in the clinical attachment at medical school.

In light of our findings, medical educators could consider the following; building knowledge base particularly around illness prognosis, exploring and encouraging positive views and enthusiasm with regards career opportunities but also ensuring that there is support and an appropriate space for students to explore any concerns such as a perceived emotional burden of the work.

We have also shown that the experience gained from a one-day visit to a specialised psychiatric unit can effect a positive change in medical students’ attitudes toward psychiatry.

Investing at the medical school stage in a doctors’ training is fundamental. Not only is there compelling evidence that the medical school psychiatry clinical attachment is vital in shaping students’ views about the speciality and the decision to pursue it as a career, but the concept of the enrichment activity outside of the traditional teaching settings could very well play an important part in this.

## Additional files

Additional file 1: Study Questionnaire. (DOC 97 kb)
Additional file 2: Study Database: DAT. database containing all study variables. (DAT 42 kb)

## Abbreviations

AMI: Attitudes to Mental Illness Questionnaire
ATP: Attitude to Psychiatry
PEAK-6: 6-item Psychiatric Experience Attitudes and Knowledge questionnaire.
