# Supplementary material for: Enrichment activities in the medical school psychiatry programme – could this be a key to engaging medical students in psychiatry? A study from a high secure forensic psychiatric UK hospital
Source: BMC Psychiatry. 2017 Mar 16;17:83. doi: 10.1186/s12888-017-1236-z (PMC5353898; doi:10.1186/s12888-017-1236-z)
Supplement: Additional file 1: — Study Questionnaire. (DOC 97 kb) [file 12888_2017_1236_MOESM1_ESM.doc]

**QUESTIONNAIRE**

**Gender** □ M □ F

**How old are you?** □ 18 - 24 □ 25 and over

**Any higher training e.g. previous degrees / qualifications prior to medical school?** □ Y □ N

**Ethnicity** Caucasian □ Asian □ Black / African / Caribbean □ Mixed □

**Experience**

During medical school: clinical placement in Psychiatry □ Y □ N

Prior to medical school: work experience / employment in mental health □ Y □ N

**Are you considering a career in Psychiatry?**

A Definitely not □ B Less likely □ C Might or might not □ D More likely □ E Definitely yes □

**Factors influencing decision** *(please tick all that apply):*

Medical school clinical placement experience □

Experience in mental health setting outside of medical school □

Perception of Psychiatry by the public □

Perception of Psychiatry by the medical profession □

Perceived lack of evidence base □

Factors related to prognosis of patients □

Job / career opportunities □

Other *(please state)*……………………………………………………………………………………..

**ATTITUDE TOWARDS PSYCHIATRY (ATP-30)** (Burra et al, 1982)

|  |  | Strongly disagree | Disagree | Neither agree or disagree | Agree | Strongly Agree |
| --- | --- | --- | --- | --- | --- | --- |
| 1 | Psychiatry is unappealing (not interesting to me) because it makes so little use of medical training. |  |  |  |  |  |
| 2 | Psychiatrists talk a lot but do very little. |  |  |  |  |  |
| 3 | Psychiatric hospitals are little more than (not very different from) prisons. |  |  |  |  |  |
| 4 | I would like to be a psychiatrist. |  |  |  |  |  |
| 5 | It is quite easy for me to accept the efficacy (effectiveness) of psychotherapy. |  |  |  |  |  |
| 6 | On the whole, people taking up psychiatric training are running away from participation in real medicine. |  |  |  |  |  |
| 7 | Psychiatrists seem to talk about nothing but sex. |  |  |  |  |  |
| 8 | The practice of psychotherapy basically is fraudulent (guilty of fraud) since there is no strong evidence that it is effective. |  |  |  |  |  |
| 9 | Psychiatric teaching increases our understanding of medical and surgical patients. |  |  |  |  |  |
| 10 | The majority of students report that their psychiatric undergraduate training has been valuable. |  |  |  |  |  |
| 11 | Psychiatry is a respected branch of medicine. |  |  |  |  |  |
| 12 | Psychiatric illness deserves at least as much attention as physical illness. |  |  |  |  |  |
| 13 | Psychiatry has very little scientific information to go on (is not based on much scientific evidence). |  |  |  |  |  |
| 14 | With the forms of therapy now (available) at hand, most psychiatric patient improve. |  |  |  |  |  |
| 15 | Psychiatrists tend to be at least as stable as the average doctor. |  |  |  |  |  |
| 16 | Psychiatric treatment causes patients to worry too much about their symptoms. |  |  |  |  |  |
| 17 | Psychiatrists get less satisfaction from their work than other specialists. |  |  |  |  |  |
| 18 | It is interesting to try to unravel (discover) the cause of a psychiatric illness. |  |  |  |  |  |
| 19 | There is very little that psychiatrists can do for their patients. |  |  |  |  |  |
| 20 | Psychiatric hospitals have a specific contribution to make to (can help specifically in) the treatment of the mentally ill. |  |  |  |  |  |
| 21 | If I were asked what I considered to be the three most exciting medical specialties, psychiatry would be excluded. |  |  |  |  |  |
| 22 | At times it is hard to think of psychiatrists as equal to other doctors. |  |  |  |  |  |
| 23 | These days, psychiatry is the most important part of the curriculum in medical schools. |  |  |  |  |  |
| 24 | Psychiatry is so unscientific that even psychiatrists can't agree as to what its basic applied sciences are. |  |  |  |  |  |
| 25 | In recent years psychiatric treatment has become quite effective. |  |  |  |  |  |
| 26 | Most of the so-called facts in psychiatry is really just vague speculations. |  |  |  |  |  |
| 27 | If we listen to them, psychiatric patients are just as human as other people. |  |  |  |  |  |
| 28 | The practice of psychiatry allows the development of really rewarding relationships with people. |  |  |  |  |  |
| 29 | Psychiatric patients are often more interesting to work with than other patients. |  |  |  |  |  |
| 30 | Psychiatry is so amorphous (vague) that it cannot really be taught effective. |  |  |  |  |  |

**QUESTIONNAIRE**

Has this visit changed your views of Broadmoor? □ Y □ N

Has this visit changed your views of Psychiatry? □ Y □ N

Has this visit made you more or less likely to take up Psychiatry as a career? □ More likely □ Less likely

**ATTITUDE TOWARDS PSYCHIATRY (ATP-30)** (Burra et al, 1982)

Please mark your level of agreement or disagreement with each statement as per the following scale:

|  |  | Strongly disagree | Disagree | Neither agree or disagree | Agree | Strongly Agree |
| --- | --- | --- | --- | --- | --- | --- |
| 1 | Psychiatry is unappealing (not interesting to me) because it makes so little use of medical training. |  |  |  |  |  |
| 2 | Psychiatrists talk a lot but do very little. |  |  |  |  |  |
| 3 | Psychiatric hospitals are little more than (not very different from) prisons. |  |  |  |  |  |
| 4 | I would like to be a psychiatrist. |  |  |  |  |  |
| 5 | It is quite easy for me to accept the efficacy (effectiveness) of psychotherapy. |  |  |  |  |  |
| 6 | On the whole, people taking up psychiatric training are running away from participation in real medicine. |  |  |  |  |  |
| 7 | Psychiatrists seem to talk about nothing but sex. |  |  |  |  |  |
| 8 | The practice of psychotherapy basically is fraudulent (guilty of fraud) since there is no strong evidence that it is effective. |  |  |  |  |  |
| 9 | Psychiatric teaching increases our understanding of medical and surgical patients. |  |  |  |  |  |
| 10 | The majority of students report that their psychiatric undergraduate training has been valuable. |  |  |  |  |  |
| 11 | Psychiatry is a respected branch of medicine. |  |  |  |  |  |
| 12 | Psychiatric illness deserves at least as much attention as physical illness. |  |  |  |  |  |
| 13 | Psychiatry has very little scientific information to go on (is not based on much scientific evidence). |  |  |  |  |  |
| 14 | With the forms of therapy now (available) at hand, most psychiatric patient improve. |  |  |  |  |  |
| 15 | Psychiatrists tend to be at least as stable as the average doctor. |  |  |  |  |  |
| 16 | Psychiatric treatment causes patients to worry too much about their symptoms. |  |  |  |  |  |
| 17 | Psychiatrists get less satisfaction from their work than other specialists. |  |  |  |  |  |
| 18 | It is interesting to try to unravel (discover) the cause of a psychiatric illness. |  |  |  |  |  |
| 19 | There is very little that psychiatrists can do for their patients. |  |  |  |  |  |
| 20 | Psychiatric hospitals have a specific contribution to make to (can help specifically in) the treatment of the mentally ill. |  |  |  |  |  |
| 21 | If I were asked what I considered to be the three most exciting medical specialties, psychiatry would be excluded. |  |  |  |  |  |
| 22 | At times it is hard to think of psychiatrists as equal to other doctors. |  |  |  |  |  |
| 23 | These days, psychiatry is the most important part of the curriculum in medical schools. |  |  |  |  |  |
| 24 | Psychiatry is so unscientific that even psychiatrists can't agree as to what its basic applied sciences are. |  |  |  |  |  |
| 25 | In recent years psychiatric treatment has become quite effective. |  |  |  |  |  |
| 26 | Most of the so-called facts in psychiatry is really just vague speculations. |  |  |  |  |  |
| 27 | If we listen to them, psychiatric patients are just as human as other people. |  |  |  |  |  |
| 28 | The practice of psychiatry allows the development of really rewarding relationships with people. |  |  |  |  |  |
| 29 | Psychiatric patients are often more interesting to work with than other patients. |  |  |  |  |  |
| 30 | Psychiatry is so amorphous (vague) that it cannot really be taught effective. |  |  |  |  |  |
